# The Clinical Effect of Oral Vitamin D2 Supplementation on Psoriasis: A Double-Blind, Randomized, Placebo-Controlled Study

**DOI:** 10.1155/2019/5237642

**Published:** 2019-04-18

**Authors:** Wareeporn Disphanurat, Wongsiya Viarasilpa, Panlop Chakkavittumrong, Padcha Pongcharoen

**Affiliations:** Dermatology Unit, Department of Internal Medicine, Faculty of Medicine, Thammasat University, Pathumthani, Thailand

## Abstract

**Background:**

There are limited randomized controlled trials of oral vitamin D supplementation in psoriasis, especially in Asia, and the results are inconclusive.

**Objective:**

To investigate the clinical effect of oral vitamin D supplementation on psoriasis.

**Methods:**

Patients with psoriasis were randomized to receive vitamin D2 60,000 IU or similar-looking placebo pills once every 2 weeks for 6 months. The primary outcome was improvement of the Psoriasis Area and Severity Index (PASI) score at 3 and 6 months after treatment. Serum levels of 25(OH)D, calcium, phosphate, parathyroid hormone, and C-reactive protein and adverse events were monitored. The chi-square test, Fisher's exact test, Student's t-test, and Spearman's correlation analysis were used in statistical analysis.

**Results:**

Of 50 subjects screened, 45 were eligible and randomized to the oral vitamin D2 group (n=23) or placebo group (n=22). At enrollment, the mean PASI score was 4.45, and 26.7% of patients had vitamin D deficiency. At 3 months, the oral vitamin D2 group had significantly higher PASI improvement than the placebo group (mean PASI improvement: 1.43 versus [vs.] -0.33, p-value=0.034; mean %PASI improvement: 34.21% vs. -1.85%, p-value=0.039). The mean serum 25(OH)D level was significantly higher in the oral vitamin D group than in the placebo group (27.4 vs. 22.4 ng/mL, p-value=0.029). Serum 25(OH)D concentrations were significantly inversely correlated with PASI scores at the 6-month follow-up. No major adverse event was observed overall.

**Conclusion:**

Oral vitamin D2 supplementation in patients with psoriasis increased the serum vitamin D level and significantly improved the treatment outcome without increasing adverse events.

**Trial Registration:**

This trial is registered with Thai Clinical Trials Registry TCTR20180613001.

## 1. Background

Psoriasis is a chronic immune-mediated inflammatory skin disease that has a complex pathogenesis. Psoriatic skin lesions result from a hyperproliferative epidermis with abnormal differentiation. The inflammatory infiltrate consists mainly of dendritic cells, macrophages, and T cells in the dermis [1, 2]. The skin serves as both a site of vitamin D biosynthesis and a target organ for vitamin D activity. Vitamin D has a role in bone and calcium metabolism, and it is important in the regulation of keratinocytes proliferation, differentiation, and apoptosis. Clinical and laboratory investigations confirmed that 1,25-dihydroxyvitamin D_3_ (1,25(OH)_2_D_3_) is effective in promoting terminal differentiation and decreasing the proliferation of cultured human keratinocytes in a dose-dependent manner [3–5]. Furthermore, vitamin D has a role in inhibiting T cell proliferation and inducing regulatory T cells [6–8]. Topical vitamin D analogs have been studied since 1985 and shown effectiveness in psoriasis; therefore, they have been used as standard therapy for decades [9, 10].

Psoriasis is a multisystem inflammatory disorder with multiple associated comorbidities, such as psoriatic arthritis, obesity, metabolic syndrome, cardiovascular, and cerebrovascular diseases [2, 6, 11–13]. Several studies showed that circulating 25 hydroxyvitamin D (25(OH)D) levels were significantly lower among patients with psoriasis than healthy controls [14–16]. Abnormal vitamin D metabolism may play a role in the pathogenesis of psoriasis [17, 18]. To date, there are limited data of the effect of vitamin D in Asian patients with psoriasis. We, therefore, conducted a study to assess its efficacy against psoriasis in Thai patients and its effect on vitamin D metabolism.

## 2. Patients and Methods

This study was approved by our local ethics committee and was registered in Thai Clinical Trials Registry, the study number TCTR20180613001. All patients gave written informed consent. The study was conducted according to the good clinical practice guideline, as well as the Declaration of Helsinki. This study was a randomized, double-blind, placebo-controlled trial which took place from November 2016 and November 2017 at Thammasat University Hospital in the Northern Bangkok Conurbation, Thailand. Fifty patients who had not responded satisfactorily to their concurrent psoriatic treatment were enrolled in the study. Forty-five patients were eligible. Inclusion criteria were patients with chronic plaque-type psoriasis, those aged 18-70 years, and those with mild psoriasis (Psoriasis Area and Severity Index [PASI] score <10). Exclusion criteria were patients currently or recently receiving systemic therapy or phototherapy within 30 days before enrollment; those with hepatic impairment, renal impairment, and cancer; those receiving immunosuppressive medication or chemotherapy, vitamin D, a calcium supplement, bisphosphonates, antiepileptic agents, and anticoagulants; those with a history of hypercalcemia, nephrolithiasis, and parathyroid disease; pregnant women; and breastfeeding women.

### 2.1. Therapeutic Regimen

Patients were randomized using a computer-generated block of four to receive oral vitamin D2 (calciferol capsules, British Dispensary, Samutprakarn, Thailand) or a placebo. The intervention was three vitamin D2 capsules (20,000 IU/capsule) every 2 weeks for 6-month duration. Participants in the placebo group received three identical-looking placebo pills every 2 weeks for 6 months. The investigators, treating physicians, and patients were all blinded to treatment allocation. There was no change in patient's current psoriatic treatment regimen other than studied intervention during the study period.

## 3. Outcomes and Follow-Up

### 3.1. Primary and Secondary Outcomes

The primary outcome was improvement of the PASI score after 3 and 6 months of continuous treatment. The secondary outcomes were the prevalence of vitamin D deficiency and insufficiency among the participants and improvement of the serum 25(OH)D concentration at 3 and 6 months after treatment. Vitamin D deficiency was defined as a serum 25(OH) vitamin D level <20 ng/ml, and vitamin D insufficiency as a serum 25(OH) vitamin D level of 21–29 ng/ml [19]. Serum 25(OH)D concentrations were evaluated by the chemiluminescence method. Changes in other laboratory parameters, including serum calcium, phosphate, parathyroid hormone, and C-reactive protein (CRP), were monitored at 3 and 6 months during the treatment period. Adverse effects were also monitored.

### 3.2. Follow-Up

At the initial visit, baseline sun exposure, dietary vitamin D intake from food, body mass index (BMI), comorbidity, and current medications including psoriasis medication were recorded by using a questionnaire. Psoriatic lesions were photographed, and the PASI scores were assessed by one dermatologist at baseline, 3 months, and 6 months. Serum levels of 25(OH)D, parathyroid hormone, calcium, phosphorus, creatinine, and CRP were recorded at baseline, 3 months, and 6 months. Clinical adverse events (AE) were sought at the follow-up visits. An AE was defined as either the appearance of a new symptom or sign or the exacerbation of a symptom or sign present at baseline. During the study, reminders by telephone were conducted fortnightly for every patient to monitor compliance and to assess for any medication changes.

## 4. Statistical Analysis

A sample size was calculated with type I error of 95% and power of 90%. The expected mean percentage of PASI change in oral vitamin D group was 50% according to Perez A. et al. study [20]. We estimated the mean percentage of PASI change in placebo group to be 25%. Considering a dropout rate of 25%, the total number of required study population was 46.

Improvements of the PASI scores in both groups were analyzed using the intention-to-treat analysis. Comparisons between mean improvements of the PASI scores in both groups were analyzed using Student's t-test, chi-square test, and Fisher's exact test, and demographic characteristics and frequencies of the side effects were compared between the groups using Student's t-test. The correlation between the serum 25(OH)D level and PASI score was determined using Spearman's rho correlation analysis. The Mantel Haenszel test was used to compare proportions within groups. All statistical analyses were performed using SPSS, version 22.0 (SPSS, Chicago, IL, USA). Statistical significance was defined as a p-value <0.05.

## 5. Results

**Figure 1:**
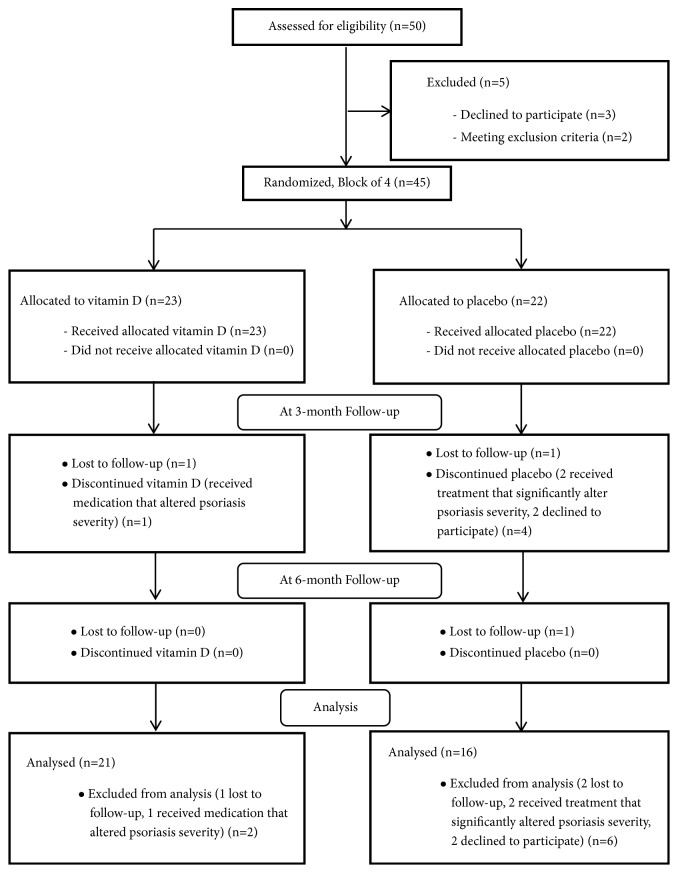
Diagram of patient recruitment.

Fifty patients were screened. Five patients were excluded, of which 3 declined to participate and 2 were taking systemic immunosuppressive agents because of their medical history. Ultimately, 45 patients were eligible and randomized to the vitamin D group (23 patients) or placebo group (22 patients) (Figure 1).

Patients' baseline characteristic and demographic data are summarized in Table 1. There was no statistically significant difference in mean age, BMI, and medication used for psoriasis between the groups. The mean baseline serum 25(OH)D levels were not statistically significantly different between the groups (vitamin D group: 24.77±5.42 ng/mL versus [vs.] placebo group: 24.13±7.74 ng/mL, p=0.75). Just over one-fourth of patients (26.7%) had vitamin D deficiency at the time of enrollment, and more than a half of patients (57.8%) had vitamin D insufficiency. The baseline PASI scores were not statistically significantly different between the groups (vitamin D: 4.68±3.12 vs. placebo group: 4.21±2.53, p=0.58).

### 5.1. Efficacy of Oral Vitamin D2 on Psoriasis

**Figure 2:**
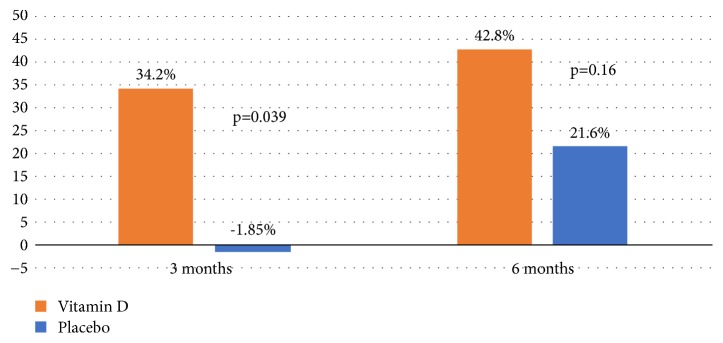
Mean percentage of Psoriasis Area and Severity Index (PASI) improvement at the 3-month and 6-month follow-ups.

At the 3-month follow-up, the mean PASI score in the vitamin D group decreased from 4.68±3.12 to 3.11±2.43, which represented a 34.21±35.24% improvement, whereas the mean PASI in placebo increased from 4.21±2.53 to 4.73±3.94, which represented a -1.85 ±66.73% worsening of the lesions (Table 2). In oral vitamin D group, 38.1% reached PASI50 and 14.3% had achieved PASI75, while in placebo group, 11.8% achieved PASI50 and 11.8% achieved PASI75, equally. The vitamin D group had significantly higher PASI improvement than the placebo group (p=0.039) (Figure 2). This improvement persisted in the patients of the vitamin D group. Two patients in the placebo group were excluded during the study period because of worsening of psoriasis. One of them required phototherapy and another needed additional medication to control the disease.

**Figure 3:**
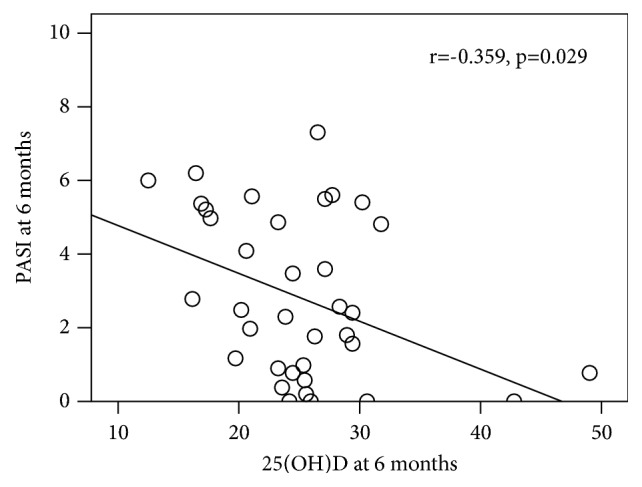
Relationship between the serum 25(OH)D level and severity of psoriasis PASI, Psoriasis Area and Severity Index.

**Figure 4:**
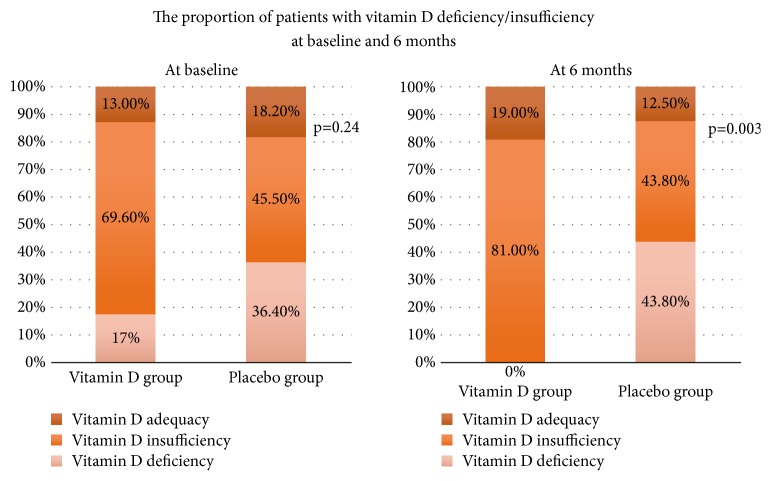
Vitamin D status in each group at baseline and the 6-month follow-up.

At the 6-month follow-up, the mean PASI score in the vitamin D group continuously decreased further to 2.39±1.97, which represented a 42.79±3.62% improvement from baseline. The mean PASI score in the placebo group decreased to 3.35±2.49, which represented a 21.57±53.22% improvement (Table 2). Although the mean PASI improvement was not statistically significantly different between the two groups at the 6-month follow-up (p=0.055), there was a trend towards higher improvement of the PASI score in the vitamin D group than in the placebo group. Among the patients in vitamin D group, 47.6% and 23.8% had clinical response to treatment and reached PASI50 and PASI75, respectively. In placebo group, 31.3% of patients achieved PASI50, and 25% of patients achieved PASI75. The 25(OH)D level was significantly and inversely correlated with the PASI score of entire study at 6 months (r=-0.359, p=0.029) (Figure 3). The proportion of patients with vitamin D deficiency in the vitamin D arm fell significantly (p≤0.001) from 17.4 to 0% whilst those in the placebo group with normal vitamin D status fell significantly (p≤0.001) from 18.2 to 12.5% (Figure 4).

### 5.2. Effect of Oral Vitamin D2 on Blood Chemistry Levels

At baseline, more than 80% of patients in each group had a serum 25(OH)D level <30 ng/ml. There was no statistically significant difference in the serum 25(OH)D level between the two groups (p=0.24). At 3 months, the mean serum 25(OH)D level in the vitamin D group was slightly increased from 24.77±5.42 ng/mL to 26.61±6.38 ng/ml. In the placebo group, the mean serum 25(OH)D level remained close to the baseline value (24.38±7.89 ng/mL). The mean 25(OH)D level was not statistically significantly different between the two groups at this follow-up. At 6 months, the mean serum 25(OH)D level was statistically significantly higher in the vitamin D group than in the placebo group (27.39±5.89 ng/mL vs. 22.44±7.28 ng/mL, p=0.029). No patient in the vitamin D group had vitamin D deficiency at the 6-month follow-up, and this was statistically significantly different when compared with the placebo group (0.0% vs. 43.8%, p=0.003) (Table 3, Figure 4).

Results of the other chemistry tests, including the parathyroid hormone, calcium, phosphorus, and CRP levels, had no statistically significant change from the baseline values. The parathyroid hormone level slightly decreased at the 6-month follow-up (baseline: 62.36±25.04 pg/ml, 3 months: 63.09±21.47 pg/ml, and 6 months: 52.651±8.87 pg/ml) in the vitamin D group, but it was not statistically significantly different between the two groups. The calcium and phosphorus levels remained close to the baseline levels in both groups during the study period. There was no report of hypercalcemia overall.

### 5.3. Adverse Events of Oral Vitamin D2 Supplementation

Overall, AEs were few. At 3-month follow-up, one patient reported drowsiness in the vitamin D group and two patients reported nausea in both vitamin D and placebo group. There were no reported AEs at the 6-month follow-up. There were no serious AEs during the study period.

## 6. Discussion

Our study demonstrated the benefit of vitamin D supplementation in psoriatic patients, as determined by the PASI score at the 3-month follow-up. This finding was consistent with that of prior studies [20–24]. We conducted a randomized, double-blind, placebo-controlled trial in a representative spectrum of Thai patients with mild psoriasis; all had PASI scores < 10. The supplementary vitamin D 60,000 IU given every two weeks had a significant benefit in Thai patients with mild psoriasis that appeared to sustain to six months. The small number of patients may have accounted for the strong trend (p=0.055) of improvement seen at 6 months. The nonsignificant difference at 6 months might be caused by the severity of psoriasis in our study, which was mild (PASI score <10). More cases of different disease severities with more inflammation may be able to exhibit a statistically significant difference. Nevertheless, we found that serum 25(OH)D concentrations were significantly inversely correlated with PASI scores of entire group at the 6-month follow-up. The higher 25(OH)D level was associated with lower severity of psoriasis.

Vitamin D has several clinical benefits in regulating bone and calcium homeostasis as well as immunomodulatory effects. There is growing evidence of the benefits of vitamin D in chronic inflammatory, autoimmune, and infectious diseases [18, 25, 26]. Previous studies of vitamin D in psoriasis have produced mixed results. An open-design study by Morimoto et al. [22] reported that 76% of patients treated orally with 1 alpha-hydroxyvitamin D3 showed significant improvement of lesions. Several studies have reported an improvement of psoriasis with oral vitamin D supplementation [20–24], but none of them was double-blind RCTs like ours. A recent RCT by Ingram et al. [27] concluded that the benefit of vitamin D3 supplementation for psoriasis could not be determined; however, they also found a significant inverse relationship between the PASI score and 25(OH)D concentration. Another RCT by Jarrett et al. [28] reported that oral vitamin D showed no significant difference in PASI compared to placebo group in mild psoriasis. Their study did not exclude patients who had changes in their psoriasis medication, the mean baseline PASI score was 3 which was lower than our patients, and the authors did not assess 25(OH)D concentration during 12-month trial, therefore unable to conclude the cause of a small improvements in PASI in both groups.

There are evidences that psoriatic patients had significantly lower serum 25(OH)D concentrations than the healthy controls [16, 29, 30] and the prevalence of vitamin D deficiency (<20 ng/ml) was significantly higher in psoriatic patients than in the controls [14]. Our study showed a higher proportion of patients with vitamin D deficiency at baseline which is almost fivefold higher than the Thai population prevalence of 5.7% [31]. These findings suggest that psoriatic patients may be at risk of vitamin D deficiency. Some studies have shown associations between vitamin D-binding protein gene polymorphism and the risk of vitamin D deficiency [32] or vitamin-D receptor gene polymorphism and the degree of response to topical vitamin D analogs [33]. The optimal dose of supplementation for inducing the immunomodulatory effect of vitamin D is still unknown and varies in many studies. We chose 60,000 IU every 2 weeks, which equates to 4,285 IU per day; this dose is within the 4,000-10,000 IU/day that is well tolerated and recommended by the Institute of Medicine and Endocrine Society recommendation [19, 34].

The limitations of our study were the small sample size and inclusion of patients with only mild psoriasis.

## 7. Conclusions

Our study demonstrated improvement of mild psoriasis with oral vitamin D2 supplementation, an increase in serum 25(OH)D concentrations, a reduced rate of vitamin D deficiency, and good tolerability. Our data suggest vitamin D2 is a good adjunctive treatment to the standard therapy. Additional studies should examine the efficacy of higher doses and longer duration of vitamin D2 in moderately severe and severe psoriasis to determine whether vitamin D would be a suitable adjunct treatment.

## Figures and Tables

**Table 1:** Baseline characteristics between the vitamin D and placebo groups.

Characteristics*∗*	Vitamin D2 N=23	Placebo N=22	p-value
*Gender*, n(%)			
Female	13(56.5%)	11(50.0%)	0.77
Male	10(43.5%)	11(50.0%)	
*Age *	52.39±14.19	49.41±15.92	0.51
*BMI*	26.30±5.20	24.9±4.78	0.36
*Fitzpatrick skin type*			1
* Type 4*	12(52.17%)	11(50%)	
* Type 5*	11(47.83%)	11(50%)	
*Underlying disease*			
Diabetes mellitus, n(%)	5(21.7%)	5(22.7%)	1.00
Hypertension, n(%)	7(30.4%)	11(50.0%)	0.23
Dyslipidemia, n(%)	7(30.4%)	6(27.3%)	1.00
*Medications*			
Tar, n(%)	17(73.9%)	19(86.4%)	0.46
Topical corticosteroids, n(%)	23(100.0%)	21(95.5%)	0.49
Topical vitamin D analogue, n(%)	14(60.9%)	13(59.1%)	1.00
Salicylic acid, n(%)	7(30.4%)	4(18.2%)	0.49
LCD, n(%)	9(39.1%)	7(31.8%)	0.76
*Sun exposure* (hour/week)	9.61±10.5	14.95±9.01	0.07
*Dietary vitamin D* (IU/week)	191.47±134.67	386.39±680.77	0.19
*PASI*	4.68±3.12	4.21±2.53	0.58
*Serum 25(OH)D level* (ng/mL)	24.77±5.42	24.13±7.74	0.75
*Vitamin D status classification * **∗** **∗**			0.24
Deficiency (<20 ng/ml), n(%)	4(17.4%)	8(36.4%)	
Insufficiency (21–29 ng/ml), n(%)	16(69.6%)	10(45.5%)	
Adequacy (>30 ng/ml), n(%)	3(13.0%)	4(18.2%)	
*Parathyroid hormone level* (pg/mL)	62.36±25.04	56.47±23.36	0.42
*Calcium level* (mg/dL)	9.03±0.36	8.94±0.36	0.40
*Phosphorus level* (mg/dL)	3.59±0.48	3.72±0.54	0.41
*CRP level* (mg/L)	6.94±6.65	3.7±4.44	0.07
*Creatinine level* (mg/dL)	0.84±0.20	0.89±0.29	0.49
*Albumin level* (mg/L)	4.04±0.43	4.11±0.24	0.51

*∗*Continuous data are mean ± standard deviation (SD); *∗∗*using the serum circulating 25-hydroxyvitamin D [25(OH)D] level, BMI: body mass index, LCD: Liquor Carbonis Detergens, PASI: Psoriasis Area and Severity Index, and CRP: C-reactive protein.

**Table 2:** Outcomes at the 3-month and 6-month follow-ups.

	Vitamin D2	Placebo	p-value
*At baseline*			
PASI, mean±SD	4.68±3.12	4.21±2.53	0.58
*At 3-month follow-up*			
PASI, mean±SD	3.11±2.43	4.73±3.94	0.13
PASI change, mean±SD	1.43±1.94	-0.33±2.95	0.03
%PASI change, mean±SD	34.21%±35.24	-1.85%±66.73	0.039
*At 6-month follow-up*			
PASI, mean±SD	2.39±1.97	3.35±2.49	0.20
PASI change, mean±SD	2.15±2.59	0.71±1.83	0.055
%PASI change, mean±SD	42.79%±36.18	21.57%±53.22	0.16

PASI, Psoriasis Area and Severity Index; SD, standard deviation.

**Table 3:** Laboratory test results at the 3-month and 6-month follow-ups.

Laboratory tests*∗*	Vitamin D2	Placebo	p-value
*At 3 months*			
Serum 25(OH)D level (ng/mL)	26.61±6.38	24.38±7.89	0.34
Vitamin D status classification			0.07
Deficiency, n(%)	2(9.5%)	7(41.2%)	
Insufficiency, n(%)	15(71.4%)	8(47.1%)	
Adequacy, n(%)	4(19.0%)	2(11.8%)	
Parathyroid hormone level (pg/mL)	63.09±21.47	55.15±22.15	0.27
Calcium level (mg/dL)	8.83±0.47	9.07±0.43	0.12
Phosphorus level (mg/dL)	3.2±0.56	3.9±1.52	0.10
CRP level (mg/L)	3.61±3.62	3.54±3.92	0.96
CRP change (mg/L)	-3.2482±6.24	-0.84±3.88	0.08
Creatinine level (mg/dL)	0.88±0.23	0.87±0.22	0.90
Albumin level (mg/L)	3.97±0.38	3.99±0.35	0.81
*At 6 months*			
Serum 25(OH)D level (ng/mL)	27.39±5.89	22.44±7.28	0.029
Vitamin D status classification			0.003
Deficiency, n(%)	0(0.0%)	7(43.8%)	
Insufficiency, n(%)	17(81.0%)	7(43.8%)	
Adequacy, n(%)	4(19.0%)	2(12.5%)	
Parathyroid hormone level (pg/mL)	52.65±18.87	49.13±13.55	0.54
Calcium level (mg/dL)	9.12±0.29	9.19±0.30	0.46
Phosphorus level (mg/dL)	3.61±0.74	3.41±0.32	0.35
CRP level (mg/L)	5.67±7.35	3.99±0.32	0.46
CRP change (mg/L)	-1.03±9.07	0.30±3.01	0.58
Creatinine level (mg/dL)	0.88±0.23	0.87±0.22	0.90
Albumin level (mg/L)	3.97±0.38	3.99±0.35	0.81

*∗*Continuous data are mean ± standard deviation (SD); CRP, C-reactive protein.

## Data Availability

All data generated or analyzed during this study are included in this published article. The datasets generated/analysed during the current study are available from the corresponding author upon reasonable request.
